# Effectiveness of real‐time visual supervised telerehabilitation on functioning following knee arthroplasty compared with outpatient or home‐based exercise: A systematic review of randomized controlled trials

**DOI:** 10.1002/jeo2.70575

**Published:** 2026-06-15

**Authors:** Alessandro de Sire, Emanuele Prestifilippo, Erminia Cofano, Claudio Curci, Nicola Marotta, Raffaella Russo, Marco Invernizzi, Antonio Ammendolia, Giorgio Gasparini, Filippo Familiari

**Affiliations:** ^1^ Physical Medicine and Rehabilitation, Department of Medical and Surgical Sciences University of Catanzaro ‘Magna Graecia’ Catanzaro Italy; ^2^ Research Center on Musculoskeletal Health, MusculoSkeletalHealth@UMG University of Catanzaro ‘Magna Graecia’ Catanzaro Italy; ^3^ Orthopedics, Department of Medical and Surgical Sciences University of Catanzaro ‘Magna Graecia’ Catanzaro Italy; ^4^ Physical Medicine and Rehabilitation Unit, Department of Neurosciences ASST Carlo Poma Mantova Italy; ^5^ Physical Medicine and Rehabilitation Unit, Department of Experimental and Clinical Medicine University of Catanzaro ‘Magna Graecia’ Catanzaro Italy; ^6^ Division of Nutrition Clinic, Department of Medical and Surgical Sciences Magna Graecia University Catanzaro Italy; ^7^ Department of Health Sciences University of Eastern Piedmont ‘A. Avogadro’ Novara Italy; ^8^ Translational Medicine, Dipartimento Attività Integrate Ricerca e Innovazione (DAIRI) Azienda Ospedaliera SS. Antonio e Biagio e Cesare Arrigo Alessandria Italy

**Keywords:** postoperative rehabilitation, rehabilitation, telemedicine, telerehabilitation, total knee replacement

## Abstract

**Purpose:**

With advances in digital healthcare technologies, telerehabilitation has recently been utilized to deliver rehabilitation in real time following orthopaedic surgery; in this scenario, therapeutic exercise, robotic rehabilitation and virtual reality approaches might be tailored to the patient's needs by professionals, utilizing digital smart devices, in a videoconferencing‐based approach with a positive impact on the patients. There remains a relative paucity of high‐quality research evaluating the effectiveness of real‐time visual‐supervised telerehabilitation (RTVST) compared to outpatient rehabilitation following total knee arthroplasty (TKA) in the current literature. This systematic review of randomized controlled trials (RCTs) aimed to assess the clinical efficacy of RTVST after TKA in terms of outcomes and knee function using the Western Ontario and McMaster Universities Arthritis Index (WOMAC) compared with outpatient or home‐based exercise.

**Methods:**

PubMed, Scopus and Web of Science databases were systematically searched for English‐language RCTs published from 2015 to 2025. Of 577 studies identified, five RCTs were included after screening according to Preferred Reporting Items for Systematic Reviews and Meta‐Analyses (PRISMA) guidelines.

**Results:**

The included studies, conducted across various countries and utilizing diverse telerehabilitation models (e.g., videoconferencing and wearable sensors), consistently suggest that RTVST is a feasible, safe and often noninferior alternative to traditional rehabilitation, with some studies indicating superior or faster functional recovery in specific parameters. RTVST also offered potential benefits in cost‐effectiveness. However, limitations such as technological barriers for the elderly and an overall high risk of bias in the included studies were noted.

**Conclusions:**

This systematic review demonstrated that RTVST provides comparable functional outcomes and patient‐reported improvements to conventional rehabilitation but highlighted the urgent need for further high‐quality research with larger sample sizes and standardized telerehabilitation protocols to clarify its precise role and optimize technological applications.

**Level of Evidence:**

N/A.

## Graphical abstract

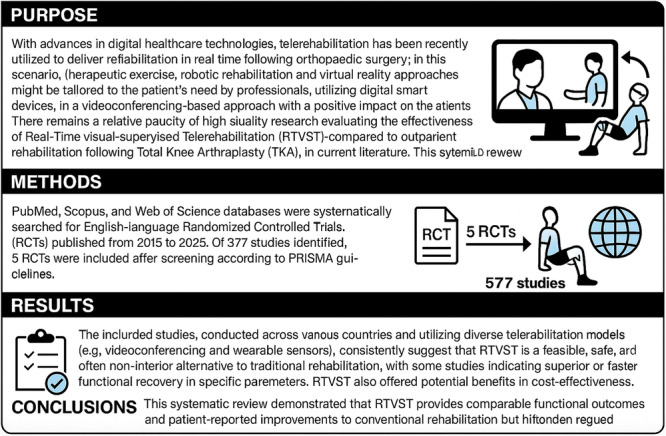

**Abbreviations**
5xsst: five times sit‐to‐stand test
IMU: inertial motion sensor
IVT: interactive virtual telerehabilitation
KOA: knee osteoarthritis
QoL: quality of life
RCTs: randomized controlled trials
ROM: range of motion
RTVST: real‐time visual‐supervised telerehabilitation
SLST: single‐leg stance test
TKA: total knee arthroplasty
WOMAC: Western Ontario and McMaster Universities Arthritis Index

## INTRODUCTION

Knee osteoarthritis (KOA) is one of the most common musculoskeletal diseases characterized by progressive degeneration of articular cartilage, subchondral bone remodelling, synovial inflammation and neuromuscular impairment [22], and it is considered an increasing socioeconomic burden in terms of medical management, cost and disability [11]. KOA primarily affects elderly women, with obesity and a sedentary lifestyle being key risk factors [7]. The global prevalence of KOA is estimated to be around 20%; specifically, in the USA, the incidence has been reported as approximately 203 cases per 10,000 person‐years in individuals aged 20 years and older [17]. One of the most effective approaches to reducing pain and restoring function is total knee arthroplasty (TKA) for end stage of KOA; this surgical procedure has shown a good long‐term outcome, patient satisfaction and cost‐effectiveness [9]. In this scenario, it is expected that the demand for TKA will increase by 673% by 2030 [9, 37].

Following TKA, physical rehabilitation is commonly provided to patients to enhance postoperative recovery, including pain reduction and improvement in muscle strength and functioning. Rehabilitation constitutes a multifaceted intervention that can be delivered at varying frequencies and intensities, across diverse settings, and is often tailored to individual social, financial and clinical circumstances, as well as responses to surgery and rehabilitation [13]. Early initiation of rehabilitation following TKA is critical to prevent complications such as joint stiffness, thromboembolism and muscle atrophy. Timely intervention also enhances gait recovery, proprioception and long‐term joint functionality [1].

To reduce postoperative limitations and enhance patient satisfaction, postoperative rehabilitation requires adequate patient education, strict compliance with treatment, and, in some cases, psychosocial support [18, 19]. While outpatient rehabilitation is commonly preferred, it may be hindered by long waiting lists, accessibility factors and high costs for both patients and health systems. This is particularly true for patients with reduced mobility, limited access to transportation or residents in areas far from the closest rehabilitation centre [5].

Patient selection for telerehabilitation is crucial, as severe sensory impairments, significant cognitive impairment, unstable medical conditions or poor balance might benefit more from inpatient rehabilitation and are generally considered contraindications to a telehealth approach [38].

In this context, telerehabilitation might play a role in allowing patients to recover in their home environment, reducing travel‐related burdens [15]. Furthermore, it has demonstrated comparable clinical effectiveness to in‐person therapy across various conditions, potentially improving patient adherence and satisfaction due to its convenience and more personalized approach compared to home‐based unsupervised exercises [21]. Therefore, in recent times, digital technologies have been proposed as a new model of rehabilitation delivery. Robotic recording devices, wearable sensors, monitors and cameras might be utilized to deliver appropriate rehabilitation in these patients with precision, and various types of telerehabilitation treatments with several intensities and durations have been reported [26].

However, most reviews included different types of interventions, such as asynchronous interviews, teleconsultations, self‐directed exercises, telephone‐based interventions or studies not specifying a telerehabilitation pathway [39, 40].

Asynchronous rehabilitation might be more flexible than real‐time rehabilitation; however, in musculoskeletal painful conditions, the latter has proved to be superior in reducing pain and disability and improving quality of life (QoL) [30, 35]. These results were also confirmed in neurological rehabilitation, with, in addition, a higher rate of adherence and successful exercise completion [6, 33]. Feedback in real time might also encourage patients to be more active in rehabilitation, and increase the intensity of the activity, while direct observation allows more immediate reporting of any adverse events during practice [32].

During the COVID‐19 era, the emerging role of telerehabilitation increased, as did its impact in monitoring patients in real time; in this scenario, therapeutic exercise, robotic rehabilitation and virtual reality approaches might be tailored to the patient's needs by professionals, utilizing digital smart devices, in a videoconferencing‐based approach with a positive impact on the patients [2, 3, 11, 32, 36].

Despite its promising advantages, there is a relative paucity of research evaluating the effectiveness of real‐time visual‐supervised telerehabilitation on functioning versus outpatient rehabilitation following TKA. In recent times, there is a need to evaluate the feasibility, acceptability and effectiveness of technological rehabilitation approaches in order to adapt to the patients' needs, potentially improving the quality of the rehabilitation in the future.

Therefore, this systematic review of randomized controlled trials (RCTs) aimed to assess the effectiveness of real‐time visual‐supervised telerehabilitation after TKA in terms of outcomes and knee functioning.

## METHODS

### Search strategy

PubMed, Scopus and Web of Science databases were systematically searched for English‐language RCTs published in the last fifteen years (from 2010 to 2025), according to a specific search string, as reported in Table 1. This systematic review was conducted according to the Preferred Reporting Items for Systematic Reviews and Meta‐Analyses (PRISMA) guidelines [24]. This systematic review was also registered on the International Prospective Register of Systematic Reviews.

**Table 1:** Systematic review specific search strings.

Electronic database	Search terms	Results
PubMed	(‘supervised exercise’ OR ‘internet‐delivered exercise’ OR ‘Telerehabilitation’) AND (‘Knee Arthroplasty’ OR ‘Arthroplasty, Knee Replacement’ OR ‘Knee Replacement Arthroplasties’ OR ‘Knee Replacement Arthroplasty’ AND (‘knee functioning’ OR ‘knee function’ OR ‘WOMAC’ OR ‘Western Ontario and McMaster Universities Osteoarthritis Index’)	249
	Filtered from 2010–2025 and RCT	
Scopus	TITLE‐ABS‐KEY ((‘supervised exercise’ OR ‘internet‐delivered exercise’ OR ‘Telerehabilitation’) AND (‘ Knee Arthroplasty’ OR ‘Arthroplasty Knee Replacement’ OR ‘Knee Replacement Arthroplasties’ OR ‘Knee Replacement Arthroplasty’) AND (‘knee functioning’ OR ‘knee function’ OR ‘WOMAC’ OR ‘Western Ontario and McMaster Universities Osteoarthritis Index’)) AND PUBYEAR > 2010 AND PUBYEAR < 2025	298
Web of Science	(‘supervised exercise’ OR ‘internet‐delivered exercise’ OR ‘Telerehabilitation’) AND (‘Knee Arthroplasty’ OR ‘Arthroplasty, Knee Replacement’ OR ‘Knee Replacement Arthroplasties’ OR ‘Knee Replacement Arthroplasty’) AND (‘knee functioning’ OR ‘knee function’ OR ‘WOMAC’ OR ‘Western Ontario and McMaster Universities Osteoarthritis Index’)	30

### Selection criteria

After the initial selection, two reviewers (C. C. and E. C.) independently screened all articles for eligibility. In case of disagreement, a consensus was reached with the opinion of a third reviewer (E. P.). The articles were selected based on the following PICO model:

(P) Participants: adults with primary TKA surgery.

(I) Intervention: supervised exercise programmes in real‐time visual‐supervised telerehabilitation via internet, sensors or app, to evaluate the patient's compliance, providing visual feedback to the therapist.

(C) Comparator: exercises delivered in a regimen of an outpatient rehabilitation setting or as home‐based rehabilitation.

(O) Outcome measure: knee pain and function, using the Western Ontario and McMaster Universities Arthritis Index (WOMAC).

Only RCTs with two groups were included (study group and control group), written in the English language. Studies with complete data were included in the final analysis, while those with missing outcomes were excluded during the screening stage.

Studies including telephone‐based interviews or any type of supervision not in real time or excluding the visual feedback were excluded; full‐text unavailability (i.e., posters and conference abstracts); reviews, case reports, articles without outcomes or results, technical notes, editorials, letters to the editor and expert opinions were excluded from the analysis.

Although the WOMAC score was considered the primary outcome due to its consistency and validity across trials, other clinically relevant endpoints, such as range of motion (ROM), muscle strength, Timed Up and Go (TUG), 6‐minute walk test (6MWT), SF‐36 and cost analysis, were also extracted and summarized as secondary outcomes.

### Data extraction

Two reviewers independently extracted main data from the included studies, utilizing a customized data extraction sheet on Microsoft Excel. In case of disagreement, a consensus was obtained by consulting another reviewer.

We extracted the following data: (1) first author; (2) publication year; (3) nationality; (4) age of study participants; (5) population and the number of patients included; (6) type of intervention, with particular of visual feedback and instrument utilized to achieve real‐time visual‐supervised telerehabilitation; (7) type of control treatment, outpatient therapeutic exercise administration; (8) knee functioning as outcome measure and (9) main findings.

### Risk of bias assessment

The methodological quality of the included RCTs was assessed using the revised Cochrane Risk of Bias tool for randomized trials (RoB 2). Two reviewers independently evaluated each study across the five domains of bias (randomization process, deviations from intended interventions, missing outcome data, measurement of the outcome and selection of the reported result). Discrepancies were resolved by consensus or with the involvement of a third reviewer.

## RESULTS

### Study characteristics

At the end of the search, a total of 577 studies were identified, 541 of which were considered suitable for title and abstract screening, after the first removal of duplicates; thus, 443 records were excluded after the title and abstract screening, according to the PICO model. Consequently, 98 articles were assessed for eligibility, and after the retrieval, 23 were excluded at the second removal of duplicates. This unusually high number of duplicates in the full‐text stage was due to metadata inconsistencies across databases (PubMed, Scopus, Web of Science), where some records were indexed differently (missing DOI, different ISBN or incomplete citation data).

**Figure 1:**
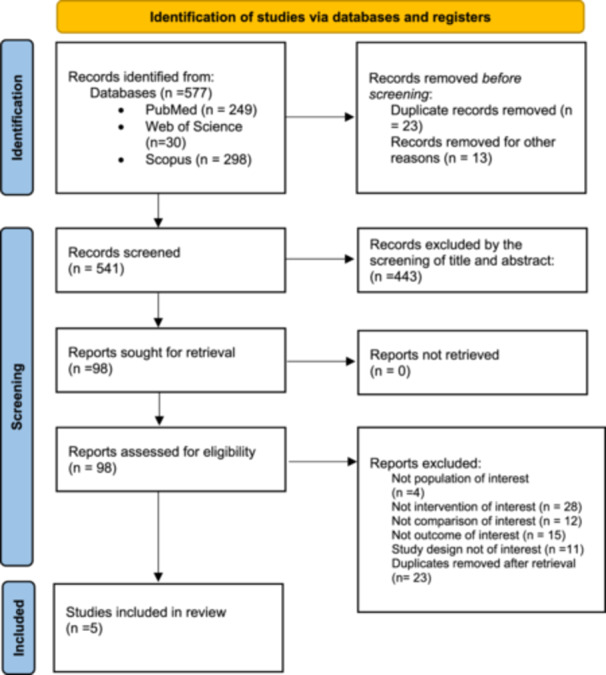
Study flowchart. PRISMA 2020 flow diagram for new systematic reviews, which included searches of databases and registers only.

In the full‐text screening, 75 studies were analysed, and 70 were excluded for the following reasons: study types different from two‐group RCT (*n* = 11), not population of interest (*n* = 4), not intervention of interest (*n* = 28), not comparison of interest (*n* = 12) and not outcome of interest (*n* = 15). Therefore, five RCTs [22, 24, 26, 27, 41] were included in this systematic review, as depicted by the PRISMA flowchart in Figure 1. The included studies have been published between 2011 [28] and 2024 [25], with a total of 298 subjects included in the telerehabilitation group and 298 included in the control group. All included trials provided complete outcome data, in accordance with our prespecified exclusion of studies with incomplete datasets. Study cohorts of the RCTs included ranged from 205 [23] to 45 [25] patients, with a mean age ranging from 65 ± 8 [41] to 68.2 ± 5.4 years [25]. Across the studies, the sex distribution varied, with most samples showing a higher proportion of female participants, reflecting the epidemiology of KOA. These studies, conducted across diverse countries including Australia, Canada, Spain, South Korea and China, evaluated various telerehabilitation models—from low‐bandwidth video consultations and app‐based programmes to advanced systems incorporating wearable sensors and real‐time feedback platforms. While intervention protocols varied in terms of duration, frequency and technological complexity, all studies assessed outcomes using standardized clinical measures such as the WOMAC index, knee ROM, muscle strength, pain scales and functional tests. When considering the magnitude of improvements, the between‐group differences observed in functional outcomes such as muscle strength and mobility were below the minimal clinically important difference (MCID) thresholds reported in the literature, supporting the interpretation that these statistical variations were not clinically meaningful. Despite the heterogeneity in intervention duration, technological platforms and supervision modalities, all studies consistently reported comparable gains in functional and quality‐of‐life outcomes, reinforcing the robustness of the findings that follow. More details of the studies' characteristics and telerehabilitation interventions are shown in Tables 2 and 3.

**Table 2:** Characteristics of the included studies.

Author (year)	Country	*N* (TR/control)	Sex (M/F)	Mode of real‐time visual supervision
Russell et al. (2011) [26]	Australia	31/34	Not reported (mean age 68 ± 7.9)	Low‐bandwidth videoconference (18 Kb/s)
Moffet et al. (2015) [22]	Canada	101/96	80 M/117 F	Interactive video platform (live therapist guidance)
Piqueras et al. (2013) [25]	Spain	90/91	84 M/97 F	Wearable sensors + 3D avatar with real‐time feedback
Zhao et al. (2024) [31]	China	50/50	48 M/52 F	Smartphone app + wearable sensors + live web portal
Nuevo et al. (2024) [24]	Spain	22/23	19 M/26 F	Wearable IMU sensors + rehub® platform (with daily asynchronous support)

Abbreviations: F, female; IMU, inertial motion sensor; M, male; TR, telerehabilitation; 3D, three‐dimensional.

**Table 3:** Characteristics of telerehabilitation interventions and controls in the included studies.

Author (year)	Intervention (TR)	Dose (frequency/duration)	Control	Primary outcomes	Secondary outcomes
Russell et al. (2011) [26]	Videoconference TR	1 session/week, 45 min, 6 weeks	Outpatient face‐to‐face rehab	WOMAC	ROM, strength
Moffet et al. (2015) [22]	Interactive video TR	16 sessions, 45–60 min over 2 months	Home‐based face‐to‐face visits	WOMAC	QoL, function
Piqueras et al. (2013) [25]	IVT + sensors	10 sessions (5 supervised + 5 independent)	Conventional in‐person rehab	Knee extension	Quadriceps/hamstring strength, WOMAC
Zhao et al. (2024) [31]	Mobile app + sensors	Daily home programme, 3 months	Printed home programme + outpatient follow‐up	Knee ROM	WOMAC, KSS, SF‐36, 5xSST, SLST
Nuevo et al. (2024) [24]	Rehub® + IMU sensors	4 weeks, daily, with asynchronous monitoring	Standard home exercise + 2–3 PT visits/week	WOMAC, VAS, EQ‐5D‐5L	Patient satisfaction, compliance

Abbreviations: 5xSST, five times sit‐to‐stand test; ADL, activities of daily living; EQ‐5D‐5L, EuroQol five dimensions, five levels; IMU, inertial motion sensor; IVT, interactive virtual telerehabilitation; KSS, Knee Society Score; QoL, quality of life; ROM, range of motion; SLST, single‐leg stance test; TKA, total knee arthroplasty; TR, telerehabilitation; VAS, visual analogue scale; WOMAC, Western Ontario and McMaster University.

### Audio‐visual real‐time feedback

A single‐blind, prospective, randomized controlled noninferiority trial conducted in Brisbane, Australia, comparing telerehabilitation to conventional face‐to‐face rehabilitation after TKA. Participants were randomized into two groups: the control group received in‐person outpatient therapy, and the telerehabilitation group received therapy via a low‐bandwidth (18 Kb/s) internet‐based videoconferencing system. Both groups underwent a standardized 6‐week rehabilitation programme with weekly 45‐min sessions, following an identical clinical pathway protocol. The primary outcome was the WOMAC global score. At the end of the intervention, both groups showed significant within‐group improvements (*p* < 0.01). The control group improved by 2.16 points (52.7%), and the telerehabilitation group by 3.26 points (67.6%), with no statistically significant between‐group difference (*F* = 3.11, *p* = 0.08). The one‐sided 95% upper confidence interval for the treatment difference (2.07 points) exceeded the predefined noninferiority margin (1.3 points), but overall results supported noninferiority of telerehabilitation. The study was generally well conducted, but concerns remained due to unclear details about allocation concealment and potential bias in outcome measurement and selective reporting.

Moreover, a multicenter, randomized controlled noninferiority trial was conducted across eight hospitals in Quebec, Canada. Patients scheduled for primary TKA due to osteoarthritis were randomized at hospital discharge to receive either telerehabilitation via an interactive video platform or standard face‐to‐face home physiotherapy.

Both groups received the same rehabilitation protocol: over two months. Telerehabilitation involved real‐time audiovisual communication and remote therapist guidance, while the control group received home visits. The primary outcome was the change in the WOMAC score from baseline to 4 months postdischarge. The primary outcome, represented by the WOMAC at four months, showed no significant differences between the groups (all *p* > 0.05), with confidence intervals within the noninferiority limits, confirming the substantial equivalence between the two treatments. Secondary outcomes, such as Knee Injury and Osteoarthritis Outcome Scores (KOOS) (*p* between 0.17 and 0.65), the 6MWT (*p* = 0.15), joint ROM and muscle strength (*p* > 0.20), also showed no statistically significant differences. This study showed strong methodological rigour across all domains, with no significant concerns regarding bias. Both studies reported significant within‐group improvements in knee flexion and extension, with no between‐group differences (*p* > 0.05) and similar gains in lower‐limb strength and joint stability. Patient satisfaction and adherence were notably high in the telerehabilitation groups, reflecting greater convenience and motivation. In Moffet et al., functional recovery was further confirmed by the absence of significant differences in the 6MWT (*p* = 0.15) and muscle strength (quadriceps *p* = 0.23; hamstrings *p* = 0.38), while a modest but statistically significant improvement was observed in the timed stair test (*p* = 0.04), suggesting slightly enhanced functional mobility with telerehabilitation.

### Wearable sensors guided telerehabilitation

A randomized, controlled, single‐blind trial [27] included patients undergoing TKA to receive either conventional in‐person rehabilitation or an interactive virtual telerehabilitation (IVT) programme. The IVT group used a digital platform with wireless sensors, three‐dimensional (3D) avatars and a web portal for remote monitoring.

The IVT programme consisted of rehabilitation at home using the system, which provided real‐time visual guidance and objective feedback on joint angles and repetitions. Both groups showed significant improvements in functional outcomes. Active knee extension improved more in the IVT group in the early phase (*p* = 0.045), and quadriceps strength showed significantly greater improvement at both 5 days (*p* = 0.011) and 3 months (*p* = 0.018). In the TUG test, both groups achieved similar outcomes by the end, despite the control group starting from a better baseline. No significant differences were found in hamstring strength, VAS for pain or WOMAC scores. The results suggested that IVT may be at least noninferior, and potentially superior, in some aspects. Despite proper randomization and outcome assessment, the high risk of bias was attributed to incomplete reporting of outcome data (notably, WOMAC scores) and potential selective reporting.

Furthermore, a prospective, randomized, assessor‐blinded, two‐arm controlled trial on this topic was conducted in China between September and November 2022 [41]. Patients were randomized equally into two groups: telerehabilitation and control.

The telerehabilitation group used the Vital Health Remote Rehabilitation System, including a mobile app, wearable sensors and a surgeon‐side web portal, for a 3‐month home‐based programme. The control group followed a standard printed home programme with routine outpatient follow‐ups. The primary outcome was knee ROM at 12 weeks. Secondary outcomes included WOMAC, KSS, SF‐36, Five Times Sit‐to‐Stand Test (5xsst), Single‐Leg Stance Test (SLST) and costs. At 6 weeks, the telerehabilitation group had significantly lower WOMAC scores (21.2 ± 11.4 vs. 25.9 ± 8.9, *p* = 0.036), indicating better early recovery. No significant differences were found in ROM or other secondary outcomes. Compliance in the telerehabilitation group was high (90%), with 94% patient satisfaction. While the methodology was generally appropriate, concerns arose regarding potential deviations from the intended intervention and a lack of clarity in reporting all predefined outcomes.

Lastly, a prospective, parallel‐group RCT on telerehabilitation with a sensor‐type monitoring was performed at Hospital Clínic of Barcelona, Spain [25]. Patients scheduled for fast‐track TKA were randomized (1:1) to either a control group (standard home exercise programme) or a telerehabilitation group using the rehub® digital platform.

Both groups followed an identical four‐week home exercise protocol. The TRH group used the rehub® system with real‐time biofeedback and wearable IMU sensors, supported by two home visits. In addition, physiotherapists performed asynchronous daily monitoring, reviewing the exercise data automatically recorded by the platform and providing feedback or follow‐up when necessary. The control group followed a paper‐based protocol and received two to three standard physiotherapy home visits per week. At four weeks, both groups improved significantly in WOMAC (telerehabilitation group: −26.9 ± 17.17; control group: −22.27 ± 13.82), with no significant between‐group differences (*p* = 0.647). Results support the feasibility and effectiveness of telerehabilitation with rehub®. The study demonstrated low risk of bias across all domains, with transparent reporting and proper handling of missing data.

Across all trials, significant within‐group improvements were observed in knee ROM, muscle strength and overall function, with no statistically significant differences compared to conventional physiotherapy (all *p* > 0.05). QoL also improved substantially, as demonstrated by higher SF‐36 and EQ‐5D‐5L scores, while patient adherence exceeded 90% in all interventions. The inclusion of wearable sensors, app‐based feedback and asynchronous monitoring enhanced engagement and compliance without increasing adverse events, supporting the safety and clinical equivalence of telerehabilitation compared with standard rehabilitation.

### Risk of bias

**Figure 2:**
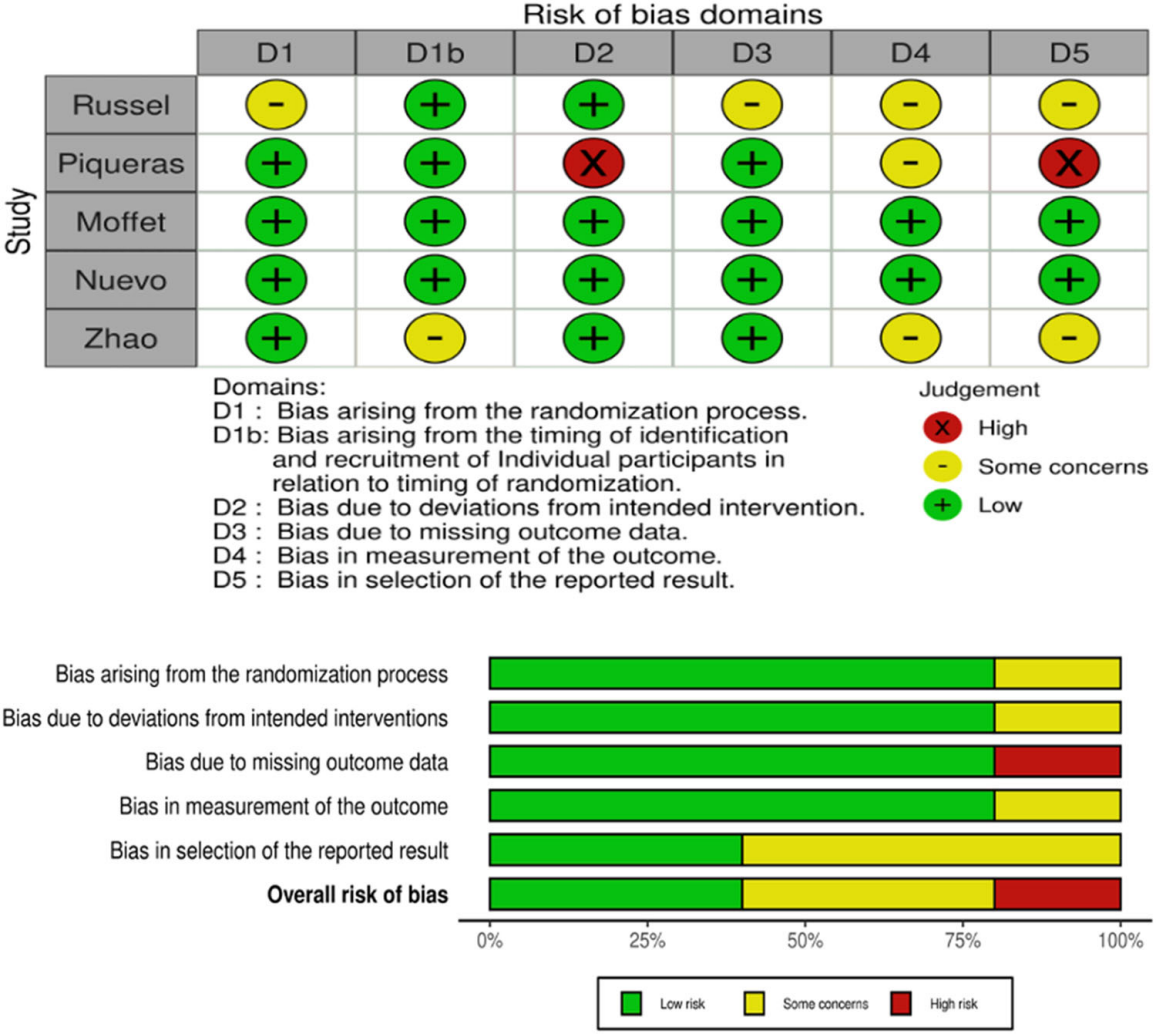
Risk of bias assessed by Version 2 of the Cochrane risk‐of‐bias tool for randomized trials (RoB 2).

Among the five included studies, one was judged to have an overall high risk of bias, two presented some concerns and two were rated as having a low overall risk of bias. Specifically, one study showed a high risk of bias due to missing outcome data, while three studies raised some concerns related to the selection of the reported results, and one showed some concern regarding the randomization process. The remaining studies demonstrated adequate methodological quality across all domains (see Figure 2).

## DISCUSSION

The purpose of this systematic review was to evaluate the existing knowledge on the clinical efficacy of real‐time visual‐supervised telerehabilitation after TKA. The findings showed that telerehabilitation with real‐time visual supervision provides comparable functional outcomes and patient‐reported improvements compared with conventional face‐to‐face rehabilitation. No significant differences were found in WOMAC score and ROM between the groups, suggesting that real‐time visual supervision is a safe and effective alternative for postoperative recovery.

In recent years, the focus on postoperative rehabilitation has grown, aiming to find a functional model that would allow the patient to achieve better results without having to face further rehabilitation admissions. In the event of epidemic outbreaks, such as COVID‐19, it may be difficult to seek clinic‐based rehabilitation. Consequently, telerehabilitation has become a viable alternative [8]. This method offers patients flexible rehabilitation options, eliminating the need for travel while providing personalized care; furthermore, there has been remarkable progress in recent years in integrating digital smart devices [5].

Of the five included RCTs, two analysed the use of rehabilitation with real‐time audio‐visual feedback. Russel et al. found that the clinical improvement of the WOMAC score in the telerehabilitation group was not statistically significant in comparison to the control group, but a significant difference was found in favour of the telerehabilitation intervention for the stiffness subscale indicating that the telerehabilitation was not inferior to the conventional intervention. In the same way, the study of Moffet et al. [23] found that the mean differences between the telerehabilitation and control group were close to zero and slightly in favour of the telerehabilitation group. These two studies utilized a videoconferencing system that enabled real‐time video and audio interaction between the patient and clinicians.

Regarding other types of telerehabilitation, three of the five studies used wearable sensors. Piqueras et al. [27] used a wireless sensor (WAGYRO) with a three‐axis accelerometer and two self‐powered gyroscopes; the group of Nuevo et al. used an inertial motion sensor (IMU) that incorporated an accelerometer, a gyroscope, a magnetometer and a strain gauge to measure forces exerted by resistance bands attached to the sensor. In this study, the WOMAC score improved, but the differences were not statistically significant [24, 39]. These results indicate that telerehabilitation is an excellent alternative, with clinical outcomes comparable to those obtained with conventional rehabilitation.

These findings are in line with the other systematic reviews published in the literature. Liu et al. [18], for instance, a meta‐analysis of RCTs, showed no significant differences in functional scores or ROM (ROM) between telerehabilitation and conventional therapy after TKA, while Zhang et al. [39] reported slightly better ROM improvement and a 43% cost‐effectiveness advantage in favour of telerehabilitation. These results confirm that telerehabilitation programmes can provide clinical outcomes equivalent to those achieved through traditional in‐person therapy. The included studies evaluated other scores; Piqueras et al. [27] observed that the muscle strength in the study group (IVT group) had a score 3 kg higher than the control group; similarly, Nuevo et al. [25] reported comparable results. This improvement may be attributed to higher adherence to the prescribed exercise plan facilitated by remote supervision and feedback. Indeed, telerehabilitation provides room for real‐time observation through digital media, encouraging patient engagement and tailored exercise progression.

This aspect is particularly crucial considering the heterogeneity of recovery courses following TKA, where initial mobilization and adherence to rehabilitation protocols are critical for optimal outcomes. These results support the integration of real‐time visual supervised telerehabilitation into postoperative care, potentially reducing healthcare system costs while maintaining quality of care, although more evidence is needed to standardize this treatment

Not all patients are ideal candidates for real‐time telerehabilitation after TKA. Factors such as cognitive impairment, low digital literacy or limited access to reliable internet connections may reduce participation and effectiveness. Clinicians should evaluate the patient's home environment, postoperative conditions and capacity to use technology before recommending remote rehabilitation. For certain patients, hybrid programmes combining remote and in‐person sessions may represent a safer and more inclusive approach.

The term ‘standardized telerehabilitation’ refers to a structured, protocol‐based intervention that uses validated clinical pathways, predefined exercise progressions and real‐time visual feedback provided by qualified therapists. Conversely, unsupervised or asynchronous telerehabilitation programmes, which rely on self‐directed exercises without live therapist interaction, may be inadequate for patients recovering from TKA, as they do not allow immediate correction of movement errors or real‐time motivation [15, 29].

Even though real‐time telerehabilitation can reduce the need for travel and resource utilization, periodic in‐person evaluations remain crucial. They allow clinicians to verify wound healing, ROM and muscle strength and to adapt exercise programmes based on individual progress and patient goals [4, 27]. Regular communication between patients and therapists also helps ensure safety and adherence throughout the recovery process [29].

Telerehabilitation provides an opportunity for real‐time observation through digital media, encouraging patient engagement and tailored exercise progression. This is particularly crucial considering the heterogeneity of recovery trajectories following TKA, where early mobilization and adherence to rehabilitation protocols are critical for optimal outcomes [34]. These findings support the integration of real‐time visual‐supervised telerehabilitation into standard postoperative care, which could potentially reduce healthcare system costs while maintaining quality of care [33].

Nevertheless, some constraints were noted. The elderly's limited familiarity with technology, technical issues and occasional lack of practical feedback could affect the overall effectiveness of telerehabilitation. These barriers have been described across the included studies, identifying digital literacy and connectivity as key limiting factors for some patient groups. Although some of the included RCTs were large and methodologically rigorous, the main limitation of the current evidence lies in the heterogeneity of intervention protocols, particularly regarding the degree of real‐time supervision, the technology used and the frequency of follow‐up. This variability makes it difficult to identify which specific telerehabilitation models are most effective after TKA. Therefore, future studies should aim to establish standardized, reproducible protocols that define optimal technical settings (e.g., real‐time video supervision, frequency of in‐person assessments) and patient selection criteria, rather than merely increasing sample size.

The risk of bias in this systematic review showed that the overall risk of bias is high, hindering the strength of the evidence. These findings demonstrated that, to date, the scientific literature should be improved on this topic. Therefore, clinicians should consider these findings when recommending telerehabilitation, weighing the delivery methods, potential benefits, the costs and the availability of alternative treatments.

Further research with larger sample sizes and standardized telerehabilitation is needed to clarify the specific conditions under which telerehabilitation might offer superior benefits against conventional rehabilitation. It is also suggested to confront the various types of telerehabilitation to find the best possible available technology to maximize the outcomes.

### Limitations of the study

Some limitations were acknowledged in this systematic review. First, only English‐language studies were considered, potentially contributing to publication bias and excluding relevant research. Second, the heterogeneity in terms of sample size and the surgical techniques used. Third, there's an ongoing debate regarding whether telerehabilitation, as a home‐based treatment, can be fully equated with supervised, face‐to‐face therapy provided in a clinical setting [14]. Fourth, the patient‐related factors such as age, motivation and home environment can significantly influence adherence to the programme. Fifth, the total sample in each treatment modality was relatively small, limiting the robustness of the conclusions. Finally, the short to mid‐term follow‐up periods with few data on long‐term functional outcomes, patient satisfaction or cost‐effectiveness.

## CONCLUSIONS

This systematic review might show real‐time visual‐supervised telerehabilitation as a promising and often comparable alternative to traditional in‐clinic rehabilitation following TKA, offering significant benefits in accessibility and cost‐effectiveness, especially given its ability to provide real‐time observation and tailored exercise progression. While existing literature, including studies using both video conferencing and wearable sensors, suggests similar clinical outcomes to conventional therapy (e.g., in WOMAC scores and ROM), the field still faces challenges such as technological barriers and a high risk of bias in current research, necessitating further rigorous studies with larger sample sizes to solidify its role and optimize its application.

## AUTHOR CONTRIBUTIONS

Alessandro de Sire and Filippo Familiari contributed to the study conception and design. Emanuele Prestifilippo, Erminia Cofano, Claudio Curci and Raffaella Russo contributed to data collection and manuscript preparation. Nicola Marotta and Marco Invernizzi were involved in data analysis and manuscript review. Antonio Ammendolia and Giorgio Gasparini provided clinical input and manuscript editing.

## CONFLICT OF INTEREST STATEMENT

The authors declare no conflicts of interest.

## ETHICS STATEMENT

The authors have nothing to report.

## Data Availability

The data that support the findings of this study are available upon reasonable request from the corresponding author.
